# ChatGPT‐4o Compared With Human Researchers in Writing Plain‐Language Summaries for Cochrane Reviews: A Blinded, Randomized Non‐Inferiority Controlled Trial

**DOI:** 10.1002/cesm.70037

**Published:** 2025-07-28

**Authors:** Dagný Halla Ágústsdóttir, Jacob Rosenberg, Jason Joe Baker

**Affiliations:** ^1^ Center for Perioperative Optimization, Department of Surgery, Herlev and Gentofte Hospital University of Copenhagen Copenhagen Denmark; ^2^ Cochrane Colorectal Group Herlev and Gentofte Hospital Herlev Denmark

**Keywords:** artificial intelligence, ChatGPT, plain language summaries, randomized controlled trial

## Abstract

**Introduction:**

Plain language summaries in Cochrane reviews are designed to present key information in a way that is understandable to individuals without a medical background. Despite Cochrane's author guidelines, these summaries often fail to achieve their intended purpose. Studies show that they are generally difficult to read and vary in their adherence to the guidelines. Artificial intelligence is increasingly used in medicine and academia, with its potential being tested in various roles. This study aimed to investigate whether ChatGPT‐4o could produce plain language summaries that are as good as the already published plain language summaries in Cochrane reviews.

**Methods:**

We conducted a randomized, single‐blinded study with a total of 36 plain language summaries: 18 human written and 18 ChatGPT‐4o generated summaries where both versions were for the same Cochrane reviews. The sample size was calculated to be 36 and each summary was evaluated four times. Each summary was reviewed twice by members of a Cochrane editorial group and twice by laypersons. The summaries were assessed in three different ways: First, all assessors evaluated the summaries for informativeness, readability, and level of detail using a Likert scale from 1 to 10. They were also asked whether they would submit the summary and whether they could identify who had written it. Second, members of a Cochrane editorial group assessed the summaries using a checklist based on Cochrane's guidelines for plain language summaries, with scores ranging from 0 to 10. Finally, the readability of the summaries was analyzed using objective tools such as Lix and Flesch‐Kincaid scores. Randomization and allocation to either ChatGPT‐4o or human written summaries were conducted using random.org's random sequence generator, and assessors were blinded to the authorship of the summaries.

**Results:**

The plain language summaries generated by ChatGPT‐4o scored 1 point higher on information (*p* < .001) and level of detail (*p* = .004), and 2 points higher on readability (*p* = .002) compared to human written summaries. Lix and Flesch‐Kincaid scores were high for both groups of summaries, though ChatGPT was slightly easier to read (*p* < .001). Assessors found it difficult to distinguish between ChatGPT and human written summaries, with only 20% correctly identifying ChatGPT generated text. ChatGPT summaries were preferred for submission compared to the human written summaries (64% vs. 36%, *p* < .001).

**Conclusion:**

ChatGPT‐4o shows promise in creating plain language summaries for Cochrane reviews at least as well as humans and in some cases slightly better. This study suggests ChatGPT‐4o's could become a tool for drafting easy‐to‐understand plain language summaries for Cochrane reviews with a quality approaching or matching human authors.

**Clinical Trial Registration and Protocol:**

Available at https://osf.io/aq6r5.

## Summary

- **What is a Plain Language Summary?**A plain language summary explains a scientific review in a simple and clear way. Cochrane reviews follow guidelines to make plain language summaries clear and easy to understand, but some still remain difficult for non‐experts. Artificial intelligence is now being tested as a way to improve their quality.
- **What Did We Want to Find Out?**We wanted to know if artificial intelligence could write plain language summaries as well as, or better than, humans and whether people could tell the difference
- **What Did We Do?**We compared 18 summaries written by humans to 18 written by artificial intelligence. Each was reviewed by four people: two experts and two non‐experts, who rated them for informativeness, readability, and level of detail. They were also asked to identify whether a plain language summary was written by a human or artificial intelligence.
- **What Did We Find?**Artificial intelligence written plain language summaries scored higher in informativeness, readability, and detail. Only 20% of reviewers correctly identified artificial intelligence‐written plain language summaries, and 64% preferred them over human‐written ones.
- This plain language summary was generated by ChatGPT and not changed in any way.

## 1 Introduction

Cochrane reviews are a rigorous collection of published research, aiming to provide an unbiased consensus on healthcare knowledge to guide physicians' and stakeholders' decisions. They are considered the foundation of evidence‐based medicine. These reviews are summarized in abstracts, but the terminology and jargon are often highly advanced and hard for the general population to read. Therefore, authors of Cochrane reviews must include a plain language summary, so that the information can reach consumers and stakeholders without a scientific background. In 80% of Cochrane reviews, their plain language summaries have been found to be inconclusive and to have a more difficult readability than the regular layperson population possesses [1, 2]. Standards exist to help authors write these plain language summaries in a uniform fashion [3] but despite this, the plain language summaries are heterogeneous and not always adherent to these standards [4].

Given the challenge of communicating Cochrane reviews to the public, artificial intelligence (AI) could be explored as a solution, such as the software ChatGPT [5]. ChatGPT predicts how a human would arrange words in a specific way in a sentence as a response to a prompt (something the user writes to get an answer), giving it the appearance of chatting. Academic writing, with its controlled environment, could be a good starting point for implementing AI in medicine. Since its debut in 2022, ChatGPT has been tested in creating abstracts [6], editing and proofreading [7], generating manuscript text [8], generating introduction sections [9], cover letters [10], and performing translations [11]. AI could, in the future, become a tool for researchers to produce better‐quality research in less time. A group of researchers has already started testing ChatGPT‐3 and found that it was satisfactory in accurately simplifying complex medical information when focusing on one paper, but inadequately synthesizing information from multiple sources [12]. Another study explored using language models to simplify clinical trial documents for general audiences with satisfactory results across most parameters [13]. Lastly, a study designed to test factual accuracies of the LLM generated PLS' found that it was usually insufficient in balancing factual accuracy and simplification of language [14].

Therefore, we hypothesized that ChatGPT‐4o was just as good as humans at writing plain language summaries for Cochrane reviews. The objective was to investigate whether ChatGPT was non‐inferior to human researchers in writing plain language summaries for Cochrane reviews.

## 2 Methods

This randomized and blinded study adheres to the CONSORT guidelines for reporting noninferiority and equivalence randomized trials [15], and CONSORT‐artificial intelligence (AI) [16] reporting guidelines. The protocol was preregistered at the Open Science Framework (OSF.io) [17]. This study was not designed to prove the superiority of AI‐generated summaries, but to explore whether layperson and expert assessors found them to be comparable or acceptable in terms of clarity, readability, and perceived quality.

We included 18 Cochrane Reviews and used their abstracts to generate new plain language summaries with ChatGPT‐4o and compared them with the plain language summary already published with the Cochrane reviews. Arm one was human written plain language summaries and the other arm was ChatGPT‐4o generated, and both were for the same Cochrane review. Data were collected in a research facility in Copenhagen, Denmark, and assessed by layman‐assessors who were recruited informally via personal networks and social media channels, and Cochrane‐assessors who were two of the co‐authors.

A prompt for ChatGPT was developed based on Cochrane's Guidance for writing a Cochrane Plain language summary [3], to generate plain language summaries. Different prompts were tested that resulted in varying levels of quality within the outputs in terms of accuracy, completeness, and tone. Several early prompts resulted in ChatGPT hallucinating, omitting content, or altering the meaning of certain items. These issues were used to guide further prompt refinements. ChatGPT was also consulted in the development of the prompt and gave input that was used further on. We created plain language summaries for two Cochrane reviews, which were tested on two of the assessors, and based on the feedback, the prompt's formatting was further adjusted. The two Cochrane reviews used during this pilot phase were later excluded from the study. The final version was selected because it consistently produced accurate, complete, and faithful translations without interpretation or errors. All history of prompt‐building was done in temporary chat mode, thus disabling ChatGPT from remembering and learning from previous prompts, which ensured that the final prompt could be used with any user of ChatGPT and not just the ChatGPT profile the prompt was built in. The 18 most recently published Cochrane reviews of interventions were extracted on the 2 December 2024. The text was inserted in a Word document where formatting was made uniform and indistinguishable between the human and the ChatGPT‐4o plain language summaries. ChatGPT‐4o was fed one abstract at a time, along with the prompt in Figure 1. Because of Open AI's memory feature in ChatGPT, the chat history was turned off to remove the risk of leaking information between the different generations of plain language summaries. Each response (e.g. plain language summary) to our prompt was saved outside of ChatGPT, and afterward, the whole chat was deleted. The content of the plain language summary was not changed in any way. The ChatGPT‐4o generated PLS were created on 2 December 2024. No summaries were generated after this date to prevent inconsistencies between updating of models. All ChatGPT‐4o generated PLS' used for this study have been compiled into a single file and can be accessed through the online repository [18]. Errors occurring from the generation of plain language summaries from abstracts were not corrected, as these were considered as an integral part of the qualities of the plain language summaries. Two Cochrane reviews' plain language summaries were excluded from the list of most recent publications during this process because they were used for pilot testing and refining the prompt and shown to the assessors before starting the trial. See the full list of included Cochrane reviews in the repository [18].

Figure 1Prompt. The full prompt given to ChatGPT‐4o after pilot‐testing and face validating, to get it to generate plain‐language summaries in the style required of the Cochrane plain‐language summary guide handbook.

**Figure:**
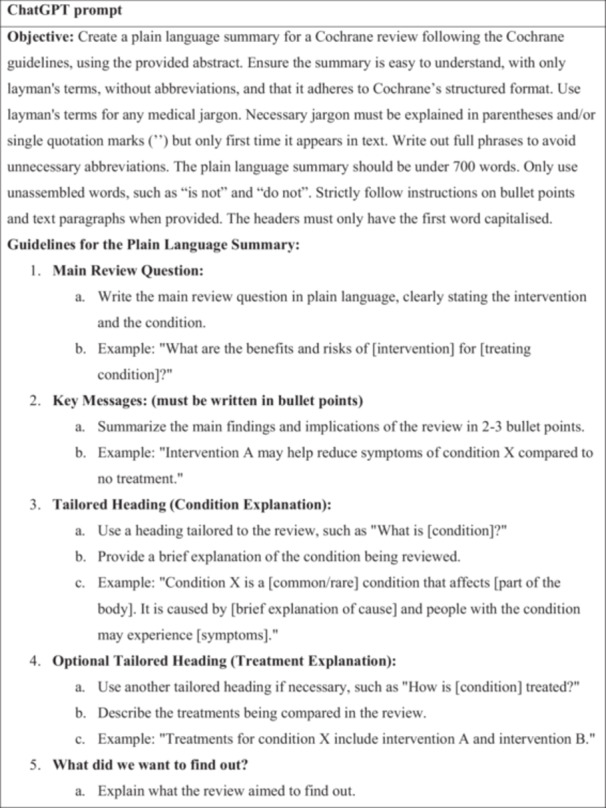

**Figure:**
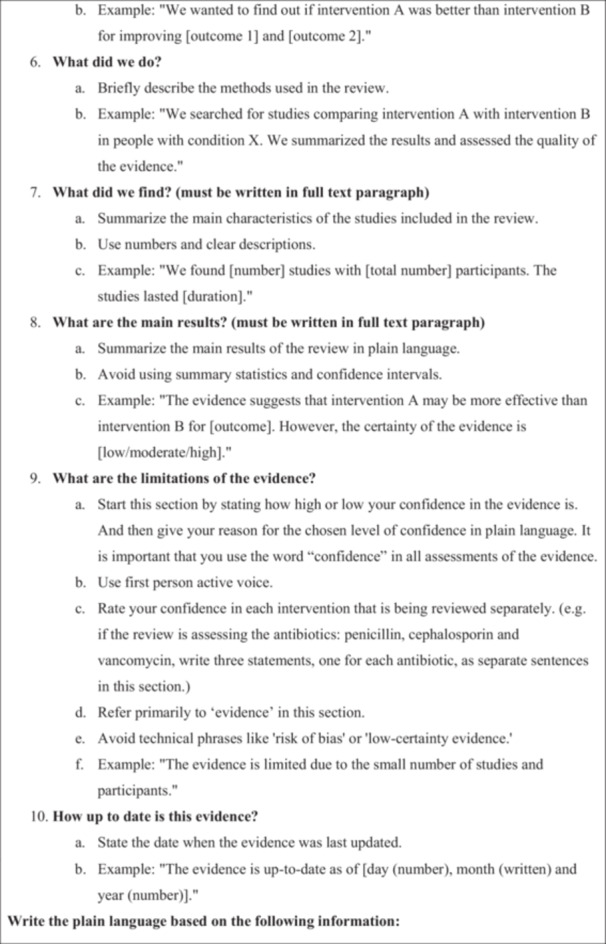

**Figure 2:**
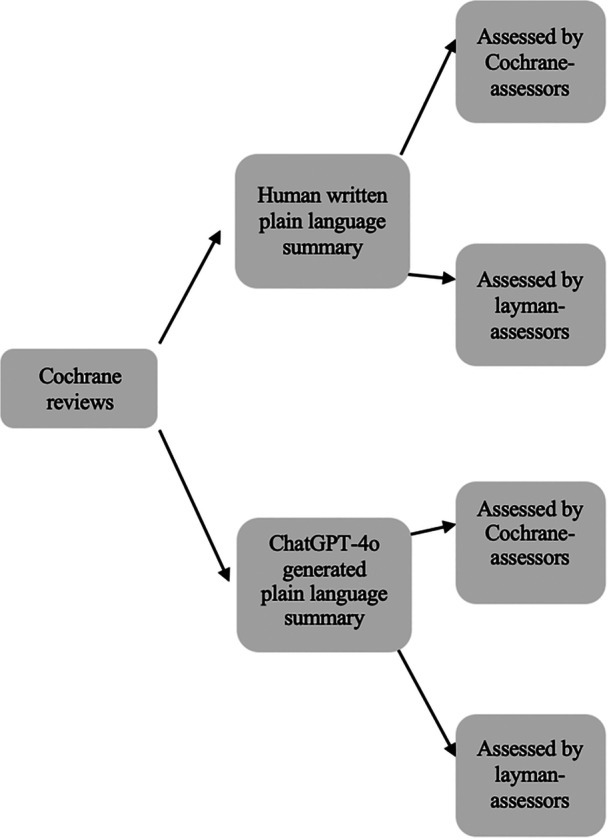
Flow diagram of plain language summary versions. Illustration of how the plain language summaries were spread and assessed. The plain language summaries were assessed with a user‐questionnaire by both layman‐assessors and Cochrane‐assessors.

The 36 plain language summaries were assessed in three different ways. They were assessed with a user‐questionnaire, which can be accessed in the original language and translated to English online [18], by two laymen (laymen‐assessors) and two members of Cochrane Colorectal's editorial group (Cochrane‐assessors) regarding the information, readability, and detail level. They were also asked whether they would submit the plain language summary as it was and guess if the plain language summary was human written or ChatGPT‐4o generated. Hence, there were 144 assessments in total with the user‐questionnaire (see flowchart in Figure 2). Secondly, they were assessed with a quality‐questionnaire [18] that was developed for the study and based on Cochrane's guidance for plain language summaries [3]. Only the Cochrane‐assessors assessed each plain language summary with both the user‐questionnaire and the quality‐questionnaire. Lastly, they were rated with the Lix [19] and Flesch‐Kincaid [20] readability scores.

In the user‐questionnaire, there were five main questions. The first three asked the assessors to rate how they were informed of the study, how easy it was to read, and if the level of detail was adequate on a Likert scale from 1 to 10. If the level of detail was not 10, that is, not perfect, then assessors were asked in a sub‐question if they thought it contained too much detail, too little detail, or a mix of both. Failing to tick a box was considered missing data, and the response was excluded from the analysis of that question. Moreover, assessors were asked to decide if they found this plain language summary good enough to be submitted with a Cochrane review, with a comment field to elaborate on their decision. The last question asked the assessors to guess if their plain language summary was ChatGPT‐4o generated or human written, with a comment field to elaborate on their decision. The user‐questionnaire was developed and validated in Danish and inspired by two similar studies [9, 10], but with questions edited for relevance. The face validation was conducted with both laypersons and healthcare professionals [17]. The quality‐questionnaire was developed from Cochrane's guidance for plain language summaries [3] and had 10 domains: template structure, title, keywords, introduction to the review, methods overview, summary of results, limitations of the evidence, currency of the evidence, use of plain language, and clarity and style. The plain language summary could get a point for fulfilling each of these (0–10 points) [18].

The readability was calculated by two calculated scores. The Lix score [19, 21], was measured by the online tool haubergs.com [22]. Lix is a readability index assessing the readability of a sentence based on the number of words divided by sentences and the number of long words divided by total words. The score is from 0 and up, with most text falling into the 20–60 point interval. Intervals 0–30 are for very easy readability, 31–40 for easy, 41–50 for medium, 51–60 for difficult, and > 60 for very difficult. The Flesh‐Kincaid score [20] was measured by the online Hemingway editor [23]. The score focuses on syllable count as a proxy for word difficulty and uses the average sentence length and average syllable count per word subtracted from the starting sum to determine the reading difficulty. In contrast to Lix, the Flesch‐Kincaid score decreases with its perceived difficulty and stratifies the scores based on grade‐level reading ability. The text difficulty scores are categorized as follows: A score of 90–100 is suitable for children and basic readers, a score of 80–89 for middle school students, 70–79 for 7th to 8th‐grade students, 60–69 for high school students, 50–59 for college students, while scores in the range of 30–49 are intended for college graduates and 0–29 is suitable for advanced readers.

The sample size was determined by calculating a significance level of 5%, power of 90%, standard deviation (SD) of outcome 1, and a non‐inferiority limit of 1, with an online tool [24]. SD was approximated based on the range in a previous similar study [10] divided by four (SD = range/4) [25].

The random allocation sequence was generated using the random.org number sequence generator. The assessors were then assigned to their summaries, in the order of the random sequence. They were thereby blinded to whether it was human written or ChatGPT‐4o generated. To prevent the risk of predictive bias, there were no restrictions on the allocation of the summaries. The human and the ChatGPT‐4o summaries were neither paired, so the summaries allocated to each assessor were completely random. No additional restrictions were applied to ensure an equal ratio of human versus ChatGPT‐4o summaries in allocation to the assessors. Thus, there was a possibility of the assessors getting five ChatGPT‐4o generated or human written summaries in a row. This was to preserve the randomness of the trial, although at the expense of exposure balance. The unique identifier number given to the reviews was only known to the first author, effectively concealing arm allocation to all assigned assessors.

Data were tested for normal distribution by assessing histograms, and all data were not normally distributed. Therefore, data were compared with the Mann–Whitney U test, and descriptive presentation was by medians and ranges. Categorical data were compared with the chi‐squared test and presented as numbers (%). The three parameters of the user questionnaire were subjected to a Bonferroni correction as the three parameters potentially influence one another. Thus, a *p*‐value for these three parameters was considered significant if it was ≤ 0.017. For all other comparisons, a *p*‐value of ≤ 0.05 was considered significant. The quality questionnaire parameters were compared for human written and ChatGPT‐4o generated PLSs with *x* ^2^‐test.

In accordance with Danish legislation, this study was exempt from requiring approval from the Danish Data Protection Agency.

## 3 Results

Data collection was from the 2 December 2024 to the 13 December 2024 (see assessment flow in Figure 2). No follow‐up was necessary as the assessments were only performed one time. There were thirteen assessors in total, and they assessed a median of six summaries each (range: 4–36): Two were Cochrane‐assessors and eleven were layperson‐assessors. To maintain objectivity, articles were selected in chronological order. The medical specialties represented in the articles included six in obstetrics and gynecology, three in cardiovascular medicine, three in neurology, and one each in child psychiatry, one stomatology/dentistry, one nephrology, one abdominal surgery, one plastic surgery, and one in healthcare organization.

ChatGPT‐4o scored significantly higher than humans on information, readability, and degree of detail (Table 1). However, the information level and degree of detail were within the non‐inferiority limit. Subjective readability was found to be significantly higher in ChatGPT‐4o generated plain language summaries than human written, with a 2‐point higher median score. Most assessors found the degree of detail satisfactory in ChatGPT‐4o generated reviews (49%), but for those who did not, they mostly found it too superficial. In human written summaries, the degree of detail level was more evenly distributed between “too detailed,” and “too superficial.” ChatGPT‐4o generated summaries were more likely to be submitted for review (64% yes) compared with human written (36% yes). Only 20% of the assessors correctly recognized/guessed summaries generated by ChatGPT‐4o, while 38% correctly identified human written summaries. The median score of the quality‐questionnaire was 1 point higher for ChatGPT‐4o generated summaries (*p* < .001), indicating that it adhered better to Cochranes guidance for plain language summaries than human written summaries. When comparing subjective ratings between Cochrane members and laypersons, we found that Cochrane members consistently provided higher scores across all three questions. Specifically, the median (interquartile range) scores for questions 1–3 were 9 (8–10), 10 (8–10), and 9 (7–9) for Cochrane members, compared to 8 (7–10), 9 (7–10), and 8 (5–10) for layperson assessors. While the trend toward higher ratings was consistent among Cochrane members, statistically significant differences between the two groups were only observed for assessments related to readability and the amount of information provided.

**Table 1:** Outcomes.

	ChatGPT	Human	*p* value
Evaluation criteria			
Information, median (range)	9 (3–10)	8 (2–10)	< 0.001
Readability, median (range)	10 (4–10)	8 (1–10)	0.002
Degree of detail, median (range)	9 (3–10)	8 (1–10)	0.004
“Too superficial”, *n* (%)	18 (26)	24 (35)	—
“Some areas too superficial others too detailed”, *n* (%)	17 (25)	18 (26)	—
“Too detailed, overall”, *n* (%)	0 (0)	10 (14)	—
“No changes needed”, *n* (%)	34 (49)	17 (25)	—
Good enough to be submitted with review, *n* (%)	44 (64)	25 (36)	< 0.001
Correct guess of who wrote it			
Correct guess, *n* (%)	14 (20)	26 (38)	0.024
Incorrect guess, *n* (%)	39 (57)	29 (42)	0.089
Would not guess, *n* (%)	16 (23)	14 (20)	0.680
Readability score			
Lix, median (range)	47 (40–55)	50 (44–57)	< 0.001
Flesch Kincaid grade level, median (range)	43 (29–61)	43 (24–57)	0.043

*Note:* The *p* values in the table are not Bonferroni corrected.

Abbreviation: *n*, number.

Half of the individual parameters were found to be fulfilled just as frequently as the human written ones, whereas the other half was found to be fulfilled significantly more often. Thus, ChatGPT‐4o more concretely followed the guidelines on writing plain language summaries compared with human authors (Table 2).

**Table 2:** Performance on quality assessment.

Quality criteria			
*N* (%) of PLSs successfully fulfilling quality criteria	ChatGPT	Human	*p* value
Follows template with headlines	36 (100)	30 (83)	0.011
Provides descriptive title	36 (100)	24 (67)	< 0.001
Satisfactory key messages	32 (89)	25 (69)	0.042
Introduces review topic and aims	36 (100)	34 (94)	0.151
Briefly explains methods in “what did we do” section	35 (97)	33 (92)	0.303
Sufficiently summarizes results in “what did we find”	34 (94)	22 (61)	< 0.001
Sufficiently describes limitations of evidence	23 (64)	13 (36)	0.018
Reports how current evidence is	35 (97)	35 (100)*	0.321
Uses simple everyday language without jargon	27 (75)	22 (61)	0.206
Adheres to style rules	35 (97)	32 (89)	0.164
Summary scores, median (range)	9 (6–10)	8 (2–10)	< 0.001

Abbreviation: *N*, number.

* Percentage differs due to one missing piece of data from the assessors questionnaire.

Objective readability measurements favor ChatGPT‐4o. Lix score (where a lower score is easier) was significantly lower for ChatGPT‐4o generated summaries, however, both are at the same level of readability called difficult reading level. Flesch‐Kincaid scores (where a higher score is easier) were higher for ChatGPT‐4o compared with humans indicating a readability of college reading level.

## 4 Discussion

We found that ChatGPT‐4o was equally as good as humans at writing plain language summaries for Cochrane reviews of interventions in regard to information level, detail level, and adherence to Cochrane guidance for writing plain language summaries when compared with human authors. The ChatGPT‐4o generated summaries performed better in the subjective assessments of readability than the human written ones. The assessors had a hard time guessing who wrote the plain language summary being assessed, with only a third correctly identifying a true ChatGPT‐4o or human written summary. In two‐thirds of assessment of ChatGPT‐4o generated summaries, they were deemed acceptable to accompany the reviews to journals, compared with only a third of the human written ones.

We found that ChatGPT‐4o was non‐inferior to humans in writing plain language summaries for Cochrane Reviews, when assessed subjectively by both Cochrane‐editorial members and laypersons. Two other studies comparing ChatGPT‐4 generated text to human written text, found that ChatGPT‐4 generated introduction sections were significantly better in terms of readability and that human written cover letters better fulfilled the criteria of content for cover letters [9, 10]. This is different from our study where we found ChatGPT to dominate in almost every aspect. In our study, the ChatGPT‐4o generated summaries scored marginally higher on the quality of content with the quality‐questionnaire. Another study from Argentina created ChatGPT generated plain‐language summaries and compared them with human written ones. Like our study, they found ChatGPT generated summaries to have a similar readability score compared with human written, but the quality of the content was not sufficient [26]. This contradicts our findings but could be due to our long and thorough prompt, which we found necessary for sufficient reporting and adherence to the Cochrane guidance for plain language summary. Other studies have found conflicting results when analyzing the quality of ChatGPT generated text. Two studies found that human written text was of significantly higher quality than ChatGPT generated text [10, 27], whereas another study found no difference between the two [9]. Several studies have investigated the readability of ChatGPT generated plain language summaries in comparison with the original abstracts. Three studies generated plain language summaries using ChatGPT by inputting professionally written abstracts. They then compared the two versions, showing that ChatGPT significantly simplified and improved reading levels compared with the original abstracts [28, 29, 30]. This aligns with our findings, that simplifying professional language with AI for improved patient health literacy is feasible. However, unlike our study, these studies did not include human written plain‐language summaries, nor did they assess the quality of its contents, which we demonstrated can be high in ChatGPT‐4o generated summaries. A feasibility study using another AI language model, Claude, created PLS from evidence reviews [31] found that accuracy, comprehensiveness, and nuances of interpretation were insufficient when AI was used compared to a human. This study differs from ours in that they compared different parameters, such as word count, which we did not do. Furthermore, they did not assess PLSs for Cochrane reviews, had a less substantial prompt and a different AI model than used in our study. Additionally, a study reported a 57% correct guess rate for human written versus 42% for ChatGPT abstracts, compared to our rates of 38% and 20%, respectively [32]. They also included AI‐detection software, which outperformed humans. Such software analyses patterns in writing (complexity, repetition, unusual phrases) by counting tokens, or the mathematical value of each word in a sentence, and calculates likelihood of it being generated by AI. This measurement was not included in our methods but could be interesting to implement in future research.

Two studies comparing ChatGPT vs human written cover letters and introduction sections found the Lix and Flesch‐Kincaid scores for ChatGPT‐4 were at a more difficult reading level when measured with text analysis tools [9, 10]. Interestingly, these objective scores did not affect the subjective readability assessments in the studies. In the case of cover letters, high difficulty of text is generally acceptable, as editors tend to have advanced reading skills. ChatGPT‐4 as a language model improves the structure and flow of text, which is not accounted for by Lix or Flesch‐Kincaid, but remains an important factor in the subjective ease of readability for human assessors. Thus, it is possible to argue that Lix and Flesch‐Kincaid scores are not a complete measure of language complexity or readers' understanding, without supplementing with a subjective readability assessment.

The strengths of this study included that it was reported according to CONsolidated Standards Of Reporting Trials (CONSORT) non‐inferiority and CONSORT‐AI. This structured approach of reporting our study ensured clarity, transparency, and reproducibility of our findings. Our study was randomized and blinded to minimize selection and preference bias and to eliminate the risk of conscious or unconscious influence of the assessors' assessment. Both subjective and objective assessments were collected to balance rigor and reliability with the real perspectives of assessors, enabling us to capture a full picture of the summaries being assessed. The user‐questionnaire was face‐validated and pilot tested, to improve the feasibility, reliability, and validity of the questionnaire. Formatting uniformly kept everything standardized, eliminating risk of assessors' assessment being affected by the format. Each summary was assessed four times by Cochrane assessors and layperson assessors. All assessors' native language was Danish, and they had to read the English summaries in their non‐native language, which reflects the real‐life scenario where the majority of consumers of academic research are non‐native English speakers. Our study also assessed the quality of content in the summaries, which was rarely, if ever done in the other studies. A limitation of our study would be that ChatGPT does not always generate the same outputs to the same prompt. To tackle this, we recommend Cochrane review authors to re‐generate prompts until they receive an appropriate output and/or edit the output to improve it even more. We intentionally refrained from doing this out of fairness to the non‐inferiority design and used the first output from ChatGPT for every prompt and abstract we fed to it. Additionally, as the language model is not open‐source and we cannot access training material, we cannot fully exclude the possibility that some of the included reviews may have already been published at the time and fed to the model as training material. Another limitation could be that recruiting informally from the network of the authors reduces the diversity of assessors, and may possibly have introduced selection bias. However, all were laypersons without any healthcare education, and are therefore the target group for plain language summaries. Subjective evaluations of the summaries by assessors may have been affected due to fatigue, as each assessor evaluated multiple summaries at a time. The answers to the first ones assessed may differ from the last ones assessed. Maturation could then also be a concern, as the assessors are likely to become better at guessing ChatGPT‐4o, the more plain language summaries they assess. Moreover, the use of a Likert scale introduces limitations, as it may not capture nuanced differences in readability or content quality between AI‐ and human‐written summaries. Such ordinal scales can lead to central tendency bias, and different assessors may interpret scale points differently. Validity of the findings in this study is limited to only the ChatGPT‐4o model, as no other versions of ChatGPT, or other types of large language models were used in the study. This is a limitation, as a new model was released while this study was being conducted [33]. Superficiality was found to be one of the key concerns identified in the ChatGPT‐4o generated text. Ideally, the writing should approach the threshold of being simplified without compromising the core message. The goal of Cochrane reviews is also to disseminate findings as broadly and effectively as possible in these plain language summaries. ChatGPT‐4o should, therefore, be trained to consistently navigate this balance, and have human oversight in medical communication. It is important to highlight that this study found that despite the goal being to improve accessibility via lower grade levels of readability, ChatGPT‐4o, like humans, was still not able to achieve that. Cochrane states that for accessibility, readability levels of PLSs should be for people without university degrees [14], so steps to further improve readability should be taken in the future. External validity could also have been affected by the relatively small sample size of only 18 pairs, but the number was calculated to be sufficient to prove non‐inferiority by a power calculation before commencing the study. Additionally, the assessors participating were both members of an editorial group in Cochrane and laypersons, making it generalizable to both these populations. Ideally, more than two Cochrane‐assessors could have participated in the study to improve the representativeness of the assessments. Furthermore, the single‐blinded design could potentially pose a limitation by introducing bias through the investigator's prior expectations and the use of Likert scales by assessors. While practical for subjective evaluation, Likert scales may lack granularity and be influenced by individual interpretation and response tendencies.

None of the other studies discussed performed an assessment of the content quality. We believe that this is an essential metric to remind us that even though readability may significantly improve with the use of large language models, it should not be at the expense of quality and factualness of outcome. We recommend future research to consider quality of content assessments. This is because we know that AI can produce wrong information, and if we are to implement AI in medicine and research, we need clear rules and guidelines on how they are to be used [30]. While ChatGPT‐4o was prompted using a customized instruction based on the Cochrane PLS guidelines, it's uncertain whether the original human‐written PLS adhered to the same guidelines. This introduces a potential methodological bias which could skew comparisons in favor of ChatGPT‐4o. Future research should establish a parallel human‐written arm given the same prompt or instructions as guidance to ensure a fair comparison. As AI gets better and increasingly popular, it still has its limitations such as data privacy, ethical responsibility, and the risk of factual inaccuracies (hallucinations). Cochrane‐level research is highly valued for its rigorous methodology but requires training and expertise. Researchers at this level value their time and prefer to focus on more important and complex tasks rather than simple and time‐consuming tasks like plain language translations. Streamlining the creation of plain language summaries with AI tools like ChatGPT could save significant time for Cochrane authors, allowing them to focus on the complex and high‐priority aspects of their work. As time spent on administrative tasks has been associated with physician burnout [34, 35, 36], reducing this burden in a research context may contribute to the improved well‐being of researchers. However, further studies are needed to explore this potential impact. Following this study, efforts to develop a custom ChatGPT tool, freely available to users with both a free and paid subscription account, that could streamline writing plain language summaries for Cochrane Reviewers are well justified. This could save much time for Cochrane authors, allowing ChatGPT to generate plain‐language summaries, and the authors to focus on other tasks.

In conclusion, this study found ChatGPT‐4o to be non‐inferior to humans in writing plain language summaries for Cochrane Reviews in terms of information level, detail level, and adherence to Cochrane guidance for plain language summaries, but with better readability. While this study suggests that ChatGPT‐4o can generate plain language summaries of comparable or in some cases higher quality than existing human‐authored summaries, further independent and large‐scale evaluations are needed. We recommend repeated experiments with a larger size to establish generalizability and consistency of these findings. Our findings highlight ChatGPT‐4o's potential to streamline plain language summary creation, while considerations about variability, factual accuracy, and ethical implications remain for future research and implementation.

## Author Contributions

**Dagný Halla Ágústsdóttir:** conceptualization, methodology, software, validation, formal analysis, investigation, resources, data curation, writing – original draft, visualization. **Jacob Rosenberg:** conceptualization, investigation, resources, writing – review and editing, visualization, supervision. **Jason Joe Baker:** conceptualization, methodology, software, formal analysis, investigation, resources, writing – review and editing, visualization, supervision.

## Ethics Statement

The authors have nothing to report.

## Consent

The authors have nothing to report.

## Conflicts of Interest

The authors declare no conflicts of interest.

## Supporting information

Supporting file 1 ‐ Abstracts list.

Supporting file 2 ‐ Questionnaire.

Supporting file 3 ‐ Critical assessment.

CESM DOI DHA.

## Data Availability

The data that support the findings of this study are available from the corresponding author upon reasonable request.
