# Supplementary material for: ChatGPT‐4o Compared With Human Researchers in Writing Plain‐Language Summaries for Cochrane Reviews: A Blinded, Randomized Non‐Inferiority Controlled Trial
Source: Cochrane Evid Synth Methods. 2025 Jul 28;3(4):e70037. doi: 10.1002/cesm.70037 (PMC12302524; doi:10.1002/cesm.70037)
Supplement: Supplementary file 1 — Supporting file 1 ‐ Abstracts list. [file CESM-3-e70037-s001.docx]

**List of included Cochrane review abstracts:**

Campisi SC, Zasowski C, Bradley-Ridout G, Schumacher A, Szatmari P, Korczak D. Omega-3 fatty acid supplementation for depression in children and adolescents. Cochrane Database Syst Rev 2024;11:CD014803.pub2. <http://doi.org/10.1002/14651858.CD014803.pub2>

Kongwattanakul K, Duangkum C, Ngamjarus C, Lumbiganon P, Cuthbert A, Weeks J, Sothornwit J. Calcium supplementation (other than for preventing or treating hypertension) for improving pregnancy and infant outcomes. Cochrane Database Syst Rev 2024;11:CD007079.pub4. <http://doi.org/10.1002/14651858.CD007079.pub4>

Annane D, Laberge L, Gallais B, Chevret S. Psychostimulants for hypersomnia (excessive daytime sleepiness) in myotonic dystrophy. Cochrane Database Syst Rev 2024;11:CD003218.pub3. <http://doi.org/10.1002/14651858.CD003218.pub3>

Zhou F, Zhang J, Li Y, Huang GQ, Li J, Wang XD. Hyaluronidase for reducing perineal trauma. Cochrane Database Syst Rev 2024;11:CD010441.pub3. <http://doi.org/10.1002/14651858.CD010441.pub3>

Rohwer C, Rohwer A, Cluver C, Ker K, Hofmeyr GJ. Tranexamic acid for preventing postpartum haemorrhage after caesarean section. Cochrane Database Syst Rev 2024;11:CD016278. <http://doi.org/10.1002/14651858.CD016278>

Worthington HV, Lewis SR, Glenny AM, Huang SS, Innes NP, O’Malley L, Riley P, Walsh T, Wong MCM, Clarkson JE, Veitz-Keenan A. Topical silver diamine fluoride (SDF) for preventing and managing dental caries in children and adults. Cochrane Database Syst Rev 2024;11:CD012718.pub2. <http://doi.org/10.1002/14651858.CD012718.pub2>

Wang Z, Zhang P, Tian J, Zhang P, Yang K, Li L. Statins for the primary prevention of venous thromboembolism. Cochrane Database Syst Rev 2024;11:CD014769.pub2. <http://doi.org/10.1002/14651858.CD014769.pub2>

Marshall MR, Wang MY, Vandal AC, Dunlop JL. Low dialysate sodium levels for chronic haemodialysis. Cochrane Database Syst Rev 2024;11:CD011204.pub3. <http://doi.org/10.1002/14651858.CD011204.pub3>

Irfan A, Rao A, Ahmed I. Single-incision versus conventional multi-incision laparoscopic appendicectomy for suspected uncomplicated appendicitis. Cochrane Database Syst Rev 2024;11:CD009022.pub3. <http://doi.org/10.1002/14651858.CD009022.pub3>

Lim AWY, Schneider L, Loy C. Galantamine for dementia due to Alzheimer’s disease and mild cognitive impairment. Cochrane Database Syst Rev 2024;10:CD001747.pub4. <http://doi.org/10.1002/14651858.CD001747.pub4>

Rocco N, Catanuto GF, Accardo G, Velotti N, Chiodini P, Cinquini M, Privitera F, Rispoli C, Nava MB. Implants versus autologous tissue flaps for breast reconstruction following mastectomy. Cochrane Database Syst Rev 2024;10:CD013821.pub2. <http://doi.org/10.1002/14651858.CD013821.pub2>

Kongwattanakul K, Pattanittum P, Jongjakapun A, Sothornwit J, Ngamjarus C, Jampathong N, Waidee T, Lumbiganon P. Prophylactic antibiotics for manual removal of retained placenta in vaginal birth. Cochrane Database Syst Rev 2024;10:CD004904.pub4. <http://doi.org/10.1002/14651858.CD004904.pub4>

Tehan PE, Mills J, Leask S, Oldmeadow C, Peterson B, Sebastian M, Chuter V. Toe-brachial index and toe systolic blood pressure for the diagnosis of peripheral arterial disease. Cochrane Database Syst Rev 2024;10:CD013783.pub2. <http://doi.org/10.1002/14651858.CD013783.pub2>

Todd CL, Johnson EE, Stewart F, Wallace SA, Bryant A, Woodward S, Norton C. Conservative, physical and surgical interventions for managing faecal incontinence and constipation in adults with central neurological diseases. Cochrane Database Syst Rev 2024;10:CD002115.pub6. <http://doi.org/10.1002/14651858.CD002115.pub6>

Mohammady M, Brown T, Radmehr M, Shamsoddin E, Janani L. Omega-3 fatty acids for intermittent claudication. Cochrane Database Syst Rev 2024;10:CD003833.pub5. <http://doi.org/10.1002/14651858.CD003833.pub5>

Shalviri G, Mohebbi N, Mirbaha F, Majdzadeh R, Yazdizadeh B, Gholami K, Grobler L, Rose CJ, Chin WY. Improving adverse drug event reporting by healthcare professionals. Cochrane Database Syst Rev 2024;10:CD012594.pub2. <http://doi.org/10.1002/14651858.CD012594.pub2>

Dwan K, Fox T, Lutje V, Lavender T, Mills TA. Perineal techniques during the second stage of labour for reducing perineal trauma and postpartum complications. Cochrane Database Syst Rev 2024;10:CD016148. <http://doi.org/10.1002/14651858.CD016148>

Sothornwit J, Ngamjarus C, Pattanittum P, Waidee T, Jampathong N, Jongjakapun A, Kongwattanakul K, Lumbiganon P. Uterotonics for management of retained placenta. Cochrane Database Syst Rev 2024;10:CD016147. <http://doi.org/10.1002/14651858.CD016147>

**List of Cochrane reviews not used due to them being used in pilot testing:**

Larkins NG, Hahn D, Liu ID, Willis NS, Craig JC, Hodson EM. Non-corticosteroid immunosuppressive medications for steroid-sensitive nephrotic syndrome in children. Cochrane Database Syst Rev 2024;11:CD002290.pub6. <http://doi.org/10.1002/14651858.CD002290.pub6>

Kiene S, Albrecht M, Theurich S, Scheid C, Skoetz N, Holtick U. Bone marrow versus peripheral blood allogeneic haematopoietic stem cell transplantation for haematological malignancies in adults. Cochrane Database Syst Rev 2024;11:CD010189.pub3. <http://doi.org/10.1002/14651858.CD010189.pub3>
