# Supplementary material for: ChatGPT‐4o Compared With Human Researchers in Writing Plain‐Language Summaries for Cochrane Reviews: A Blinded, Randomized Non‐Inferiority Controlled Trial
Source: Cochrane Evid Synth Methods. 2025 Jul 28;3(4):e70037. doi: 10.1002/cesm.70037 (PMC12302524; doi:10.1002/cesm.70037)
Supplement: Supplementary file 2 — Supporting file 2 ‐ Questionnaire. [file CESM-3-e70037-s003.pdf]

Spørgeskema til vurdering af lægmandsresuméer skrevet enten af ChatGPT-4o eller af et menneske

I Cochrane reviews af interventioner skal der foruden et abstract/resumé skrives et lægmandsresumé (plain language summary). Du bedes tage stilling til to lægmandsresuméer, hvor det ene er skrevet af et menneske og blevet publiceret som del af en videnskabelig artikel og det andet er skrevet af ChatGPT-4o.

Resumé ID (filnavn): ________

Lægmandsresumé

1.1 Formålet med lægmandsresuméer er at formidle information til ikke-sundhedsfaglige personer. Efter at have læst denne tekst, hvor godt synes du på en skala fra 1–10, at du er blevet informeret omkring studiet?

☐	☐	☐	☐	☐	☐	☐	☐	☐	☐
1<br>Dårlig	2	3	4	5	6	7	8	9	10<br>Fremrag<br>ende

1.2 Hvordan vil du vurdere den samlede læsbarhed af denne tekst på en skala fra 1–10?

☐	☐	☐	☐	☐	☐	☐	☐	☐	☐
1<br>Dårlig	2	3	4	5	6	7	8	9	10<br>Fremrag<br>ende

1.3 Hvordan var detaljegraden for dig?

☐	☐	☐	☐	☐	☐	☐	☐	☐	☐
1<br>Dårlig	2	3	4	5	6	7	8	9	10<br>Fremrag<br>ende

1.4 Hvis du valgte lavere end 10 i spørgsmål 1.4, hvorfor?

☐	☐	☐
For overfladisk	Nogle steder for overfladisk og andre steder for detaljeret	For detaljeret

2. Til sidst

2.1 Ville du indsende dette lægmandsresumé med artiklen til tidsskriftet?

☐ Ja ☐ Nej

a) Hvorfor?

2.2 Tror du at dette lægmandsresumé var skrevet af et menneske eller af ChatGPT?

Menneske	ChatGPT	Kan ikke afgøre

a) Hvorfor?
