# Supplementary material for: ChatGPT‐4o Compared With Human Researchers in Writing Plain‐Language Summaries for Cochrane Reviews: A Blinded, Randomized Non‐Inferiority Controlled Trial
Source: Cochrane Evid Synth Methods. 2025 Jul 28;3(4):e70037. doi: 10.1002/cesm.70037 (PMC12302524; doi:10.1002/cesm.70037)
Supplement: Supplementary file 3 — Supporting file 3 ‐ Critical assessment. [file CESM-3-e70037-s004.pdf]

Critical assessment of PLS according to Cochrane guidance for writing a PLS

Insert PLS ID here__________________

https://training.cochrane.org/handbook/current/guidance-writing-cochrane-plain-language-summary.pdf

Assess if the PLS fulfills each criterion (No/Yes)	Sæt kryds<br>**No**	 **Yes**
Template for Cochrane Plain language summaries		
**Does it follow the template with the following headlines? (one answer):**<br>- Plain language summary title.<br>- Key messages.<br>- Tailored heading: for example, What is epilepsy?<br>- Optional tailored heading: for example, How is epilepsy treated?<br>- What did we want to find out?<br>- What did we do?<br>- What did we find?<br>- Optional heading: Main results.<br>- What are the limitations of the evidence?<br>- How up to date is this evidence?		
Guidance: the Cochrane Plain language summary, section by section		
**Title:** Examples of text they could use:<br>o What are the benefits and risks of intervention for [treating] condition?<br>o Intervention a or intervention b: which works better to treat condition?		
**Key messages**<br>- First key message. Aim to answer the review question asked in the title, and remember to include any unwanted or harmful effects, or state that they were not reported. If the review could not answer the question in the title, state this as the first key message, with the reason why (for example, no studies found). Remember to give a sense of the quality of the evidence and state to whom the results apply.<br>- Second key message (optional). Include any other significant finding or important secondary objective.<br>- Final key message. State what should happen next. For example, future studies should measure the longer-term effects/last longer than 1 year. Mention key limitations and important unanswered questions here.		
**Introduction to the review topic and review aims**<br>Corresponds to the ‘Background’, ‘Objectives’ in the original abstract and refers to the sections “**Tailored heading (What is…)**”, “**Optional tailored heading (How is…)**”, and “**What did we want to find out?**”<br>The PLS should include a brief explanation of the review topic. It should provide enough information for the reader to understand:<br>- What the review is about.<br>- What is the condition of interest?<br>- How is it treated?<br>- What the review authors wanted to find out.		
**Brief mention of the methods**<br>This corresponds to the Methods section of the review and refers to the “**What did we do?**” in the PLS.		

<table>
<tr><td>The Plain language summary should explain the review methods very briefly. For example, that the review authors:
- Searched for studies with specific characteristics (for example, about a specific population, treatment, or comparison).
- Summarized the evidence across studies.
- Evaluated the evidence.</td><td></td><td></td></tr>
<tr><td>Summary of results
This corresponds to the Results section of the review and refers to “What did we find?” in PLS. This section should report:
- The main characteristics of the studies that were included in the review, such as number of studies and people, characteristics, follow-up, funding sources, study setting (such as country), types of intervention and comparisons.

- The main results of the review. Include unwanted and harmful effects as well as positive effects. Do not include summary statistics and confidence intervals.

- See examples in PLS guidance page 15 for suggested wording for narratives depending on different effect sizes and level of certainty of evidence.</td><td></td><td></td></tr>
<tr><td>Main limitations of the evidence
This corresponds to the quality of the evidence section in the Discussion and SOF table, and refers to the “What are the limitations of the evidence?” in the PLS. The PLS should mention the main reasons for downgrading the certainty of the evidence, using plain language.
<table>
<tr><th>GRADE judgement</th><th>Explanation of limitations of the evidence</th></tr>
<tr><td>High certainty</td><td>We are confident that …</td></tr>
<tr><td>Moderate certainty</td><td>We are moderately confident in the evidence because…
Our confidence in the evidence is only moderate because of concerns about … followed by the main reasons for downgrading the evidence.</td></tr>
<tr><td>Low certainty</td><td>We have little confidence in the evidence because … followed by the main reasons for downgrading the evidence.</td></tr>
<tr><td>Very low certainty</td><td>We are not confident in the evidence because … followed by the main reasons for downgrading the evidence.</td></tr>
</table>
See table in PLS guidance for suggested wording for reasons for up- or downgrading.</td><td></td><td></td></tr>
<tr><td>Reporting how current the evidence is
The evidence is up to date to month and year of search.</td><td></td><td></td></tr>
<tr><td><u>General advice on writing in plain language</u></td><td></td><td></td></tr>
<tr><td>Language
Use everyday language; avoid long words and research jargon; explain medical words and technical terms; avoid acronyms and abbreviations; write for the international audience.</td><td></td><td></td></tr>
<tr><td>Style
Keep paragraphs and sentences short; use active voice; use pronouns; use verbs; write numbers as numerals (1, 2, 3,…); Be concise</td><td></td><td></td></tr>
<tr><td>Summary of results (1 point for each Yes):</td><td colspan="2"></td></tr>
</table>
